# Biomaterials direct functional B cell response in a material-specific manner

**DOI:** 10.1126/sciadv.abj5830

**Published:** 2021-12-01

**Authors:** Erika M. Moore, David R. Maestas, Chris C. Cherry, Jordan A. Garcia, Hannah Y. Comeau, Locke Davenport Huyer, Sean H. Kelly, Alexis N. Peña, Richard L. Blosser, Gedge D. Rosson, Jennifer H. Elisseeff

**Affiliations:** 1 Department of Materials Science and Engineering, University of Florida, Gainesville, FL, USA.; 2 Translational Tissue Engineering Center, Wilmer Eye Institute and the Department of Biomedical Engineering, Johns Hopkins University, Baltimore, MD, USA.; 3 Bloomberg~Kimmel Institute for Cancer Immunotherapy, Sidney Kimmel Comprehensive Cancer Center, Johns Hopkins University School of Medicine, Baltimore, MD, USA.; 4 Division of Plastic Surgery, Department of Surgery, Johns Hopkins University, Baltimore, MD, USA.

## Abstract

B cells are an adaptive immune target of biomaterials development in vaccine research but, despite their role in wound healing, have not been extensively studied in regenerative medicine. To probe the role of B cells in biomaterial scaffold response, we evaluated the B cell response to biomaterial materials implanted in a muscle wound using a biological extracellular matrix (ECM), as a reference for a naturally derived material, and synthetic polyester polycaprolactone (PCL), as a reference for a synthetic material. In the local muscle tissue, small numbers of B cells are present in response to tissue injury and biomaterial implantation. The ECM materials induced mature B cells in lymph nodes and antigen presentation in the spleen. The synthetic PCL implants resulted in prolonged B cell presence in the wound and induced an antigen-presenting phenotype. In summary, the adaptive B cell immune response to biomaterial induces local, regional, and systemic immunological changes.

## Teaser

Biomaterial composition contributes to local, regional, and systemic alterations in B cells.

## INTRODUCTION

The immune system is a central target of modern-day therapeutic strategies in cancer, autoimmunity, and other diseases (*1*, *2*). It has also emerged as an important contributor to regenerative medicine and wound healing (*3*, *4*). Biomaterial-based technologies are frequently a component of regenerative medicine strategies, where they serve as a scaffold for cell proliferation and differentiation and ultimately enable previously unidentified tissue development (*5*, *6*). After implantation, biomaterials induce a foreign body response (FBR) (*7*). The immune response to biomedical implants is defined by a cascade of protein deposition, followed by neutrophil recruitment, and macrophage responses that result in fibrosis at the material interface (*7*, *8*). In the case of biomaterial scaffolds in regenerative medicine, the combined immune response to foreign biomaterial and localized tissue injury affect the functional outcomes on the spectrum of regenerative repair to fibrosis. With technological development, the opportunities to better probe the diverse immune cell phenotypes that govern biomaterial-associated host response continue to grow.

B cells, a cell type of the adaptive immune system, are primarily recognized for their ability to produce antibodies (*9*). In an effort to harness this effect, B cells have frequently been targeted with biomaterials, primarily in the form of micro- and nanoparticles, in the context of vaccine development for infection response, treatment of autoimmune disease, and cancer therapy (*5*, *10*). Other roles of B cells have received less attention in the context of the biomaterial response, including cytokine secretion, activation of other immune cells, and antigen presentation. Although B cells make up a small percentage of cell types present local to biomaterial tissue implants, their potential importance in regenerative medicine is illustrated in B cell knockout (KO) models that resulted in reduced wound healing and reduced fibrosis (*8*, *11*–*13*). Research on B cells thus far is mixed with divergent roles of B cells found in the biomaterial response and tissue repair. In one case, delivery of mature B cells promoted wound healing of skin incisions. In contrast with an alginate biomaterial, B cell presence correlated to fibrotic environments mediated through macrophage recruitment (*12*, *13*). Furthermore, reports of lymphoma development with synthetic breast implants also suggest B cell activity in detrimental fibrotic material responses (*14*–*17*). Considering that B cells traffic throughout the body, their response to biomaterials and wounds may hold important physiological implications on the systemic response to biomaterial implants.

In this work, we present an in-depth evaluation of the B cell response to biological and synthetic scaffolds in a muscle injury. This work represents two extrema of biomaterials used clinically that can more broadly serve as model systems. We characterize the kinetics and phenotype of B cells in response to a tissue extracellular matrix (ECM) biological scaffold and a synthetic polyester polycaprolactone (PCL) biomaterial implant. B cell phenotype changed in the regional lymph nodes (LNs) and spleen depending on the material composition. Biological scaffolds induced an earlier B cell presence in the muscle wound environment and germinal center formation in the draining LN. In contrast, PCL materials showed prolonged B cell presence and primarily induced a phenotype characterized by increased antigen presentation. Lack of mature B cells in a muMt^−^ KO model reduced the fibrotic response to PCL and decreased expression of inflammatory genes associated with fibrosis.

## RESULTS

### Biomaterial scaffolds alter the B cell response to injury in the local tissue and regional LNs

To characterize the B cell response to biomaterials, we performed a volumetric muscle loss (VML) injury on C57BL/6 wild-type (WT) mice and implanted two materials, submucosal-intestine ECM (SIS-ECM) or PCL. SIS-ECM promotes a pro-healing type 2 immune response characterized by interleukin-4 (IL-4) secretion (*18*, *19*), while PCL promotes a type 17 immune response characterized by IL-17 secretion that yields the generation of senescent cells and a fibrotic capsule (*20*). To assess response phenotype, we performed multiparametric flow cytometry on the injured muscle tissue with and without biomaterials and the corresponding draining LN in comparison to naïve tissue (gating strategy shown in fig. S1, A and B). The number of CD45^+^ immune cells in the wound increased with implantation of biomaterial as expected based on previous studies (fig. S2A).

To assess B cells in the implants and tissue, we evaluated B220 (CD45R) and CD19 to differentiate mature and resting B cells (*21*, *22*). B220 is a pan-B cell marker, while CD19 expression correlates to B cell developmental stage (*21*, *22*). The loss of CD19 expression is characteristic of differentiated plasma B cells (*23*). Long-lived differentiated plasma cells can be further differentiated from short-lived plasma cells by their expression of B220 and major histocompatibility complex (MHC) II (*24*). Therefore, CD19^−^B220^+^ may represent long-lived differentiated B cells (*23*–*25*). B cells in this work are defined as CD45^+^ cells expressing CD19^+^B220^−^, CD19^−^B220^+^, and CD19^+^B220^+^. For the results, each category of B cells will be referenced according to the markers used. In the muscles containing biomaterials, CD19^+^B220^−^, CD19^−^B220^+^, and CD19^+^B220^+^ B cells comprised a small percentage (3 to 12%) of the CD45^+^ cells in the muscles containing biomaterials (fig. S2B). The presence of B cells shifts as a function of time point following VML injury (fig. S2B).

**Fig. 1.**
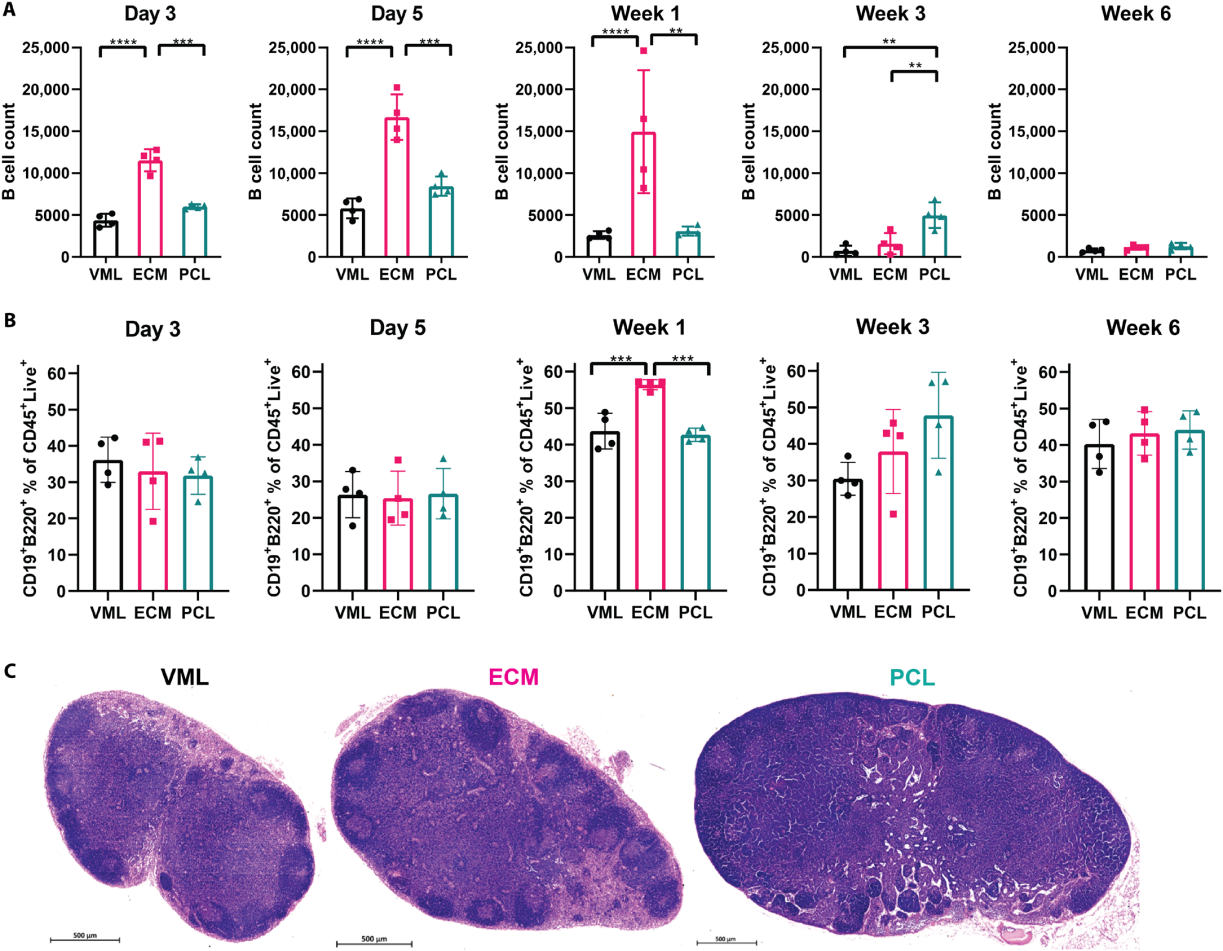
Biomaterial implants have diverging influences on B cells in quad tissue and in LN. (**A**) Flow cytometry counts of a subset of B cells (defined as CD19^−^B220^+^) in quad over time after injury. B cell number in tissue peaks at day 5 in ECM and at weeks 1 and 3 in PCL. (**B**) Flow cytometry assessment of B cells in the LN (represented as a percentage of CD45^+^Live^+^ cells) following injury and biomaterial implant. (**C**) Histology of LNs at 3 weeks after injury. Data are means ± SD, *n* = 4, two-way analysis of variance (ANOVA) with subsequent multiple comparison testing. ANOVA [(A and B): ****P < 0.0001, ***P < 0.001, **P < 0.01]. VML indicates injury with saline, ECM indicates injury implanted with ECM, and PCL indicates injury implanted with PCL.

The presence of CD19^−^B220^+^ B cell in the muscle injury depended on the presence and type of biomaterial (Fig. 1A). At day 3 after surgery, the earliest time point analyzed, the greatest number of CD19^−^B220^+^ B cells in the muscle tissue was in the SIS-ECM biomaterial group (21,570 ± 7452), compared to substantially lower numbers in the injury with saline control (9064 ± 3077). In the SIS-ECM environment, CD19^−^B220^+^ B cells peak in cell number between day 5 and 1 week after injury. In contrast, PCL-treated wounds reached peak CD19^−^B220^+^ B cell presence at day 5 and 3 weeks after injury, relative to other treatment groups. At week 3 after injury, PCL implantation resulted in more CD19^−^B220^+^ B cells (11,355 ± 1113) when compared to SIS-ECM (6501 ± 1691) and injury with saline-treated (4682 ± 1819) groups. At 6 weeks after injury, CD19^−^B220^+^ B cell numbers in the tissue were low and were similar in all treatment groups.

B cell changes were also seen in the draining inguinal LN (ILN) (Fig. 1, B and C). Injury alone increased the total numbers of CD19^+^B220^+^ B cells in the ILN (fig. S2C). The percentage of CD19^+^B220^+^ B cells in the ILN significantly increased at 1 week after injury in muscle tissue treated with ECM compared to injury with saline and PCL treatment (Fig. 1B). PCL treatment resulted in the greatest percentage CD19^+^B220^+^ B cells in the ILN at 3 weeks after injury (fig. S2D). By 6 weeks, the percentage of CD19^+^B220^+^ B cells in the ILN was similar in all groups. Histology at the 3-week time point (Fig. 1C) indicates dense circular regions on the periphery of the ILN in the ECM group compared to injury (VML) and PCL. The dark circular regions are denser, larger in area, and more distinct in the ECM sample than in the other examples.

Given the increase in CD19^+^B220^+^ B cells at the 1-week time point in ECM and the second trending increase in PCL at the 3-week time point, we further evaluated CD19^+^B220^+^ B cells using multiplex gene expression analysis of CD19^+^B220^+^ B cells at both time points. We sorted from the ILN and conducted NanoString for the 1-week time point. ECM-treated B cells differentially up-regulated B cell phenotypic genes compared to injury alone (fig. S3, full expression data in data file S1). ECM and PCL treatment also up-regulated genes associated with B cell signaling, including *Mif*, *Cd69*, and *Cd79a*, when compared to injury controls (fig. S3A). ECM treatment up-regulated expression of *Prdm1* (data file S1) that encodes the *Blimp1* gene, a regulator of immunoglobulin secretion, and generation of long-lived mature B cells (*26*). PCL treatment induced a down-regulation of *Prdm1* expression, suggesting that ECM treatment supported more generation of differentiated B cells. Both biomaterial implant conditions up-regulated antigen processing–related genes such as *Cd1d1*, *Relb1*, *Icam1*, and *H2-DMb1* in B cells (fig. S3C).

### Single-cell RNA-seq of B cells reveals differentiated B cell generation after ECM treatment

**Fig. 2.**
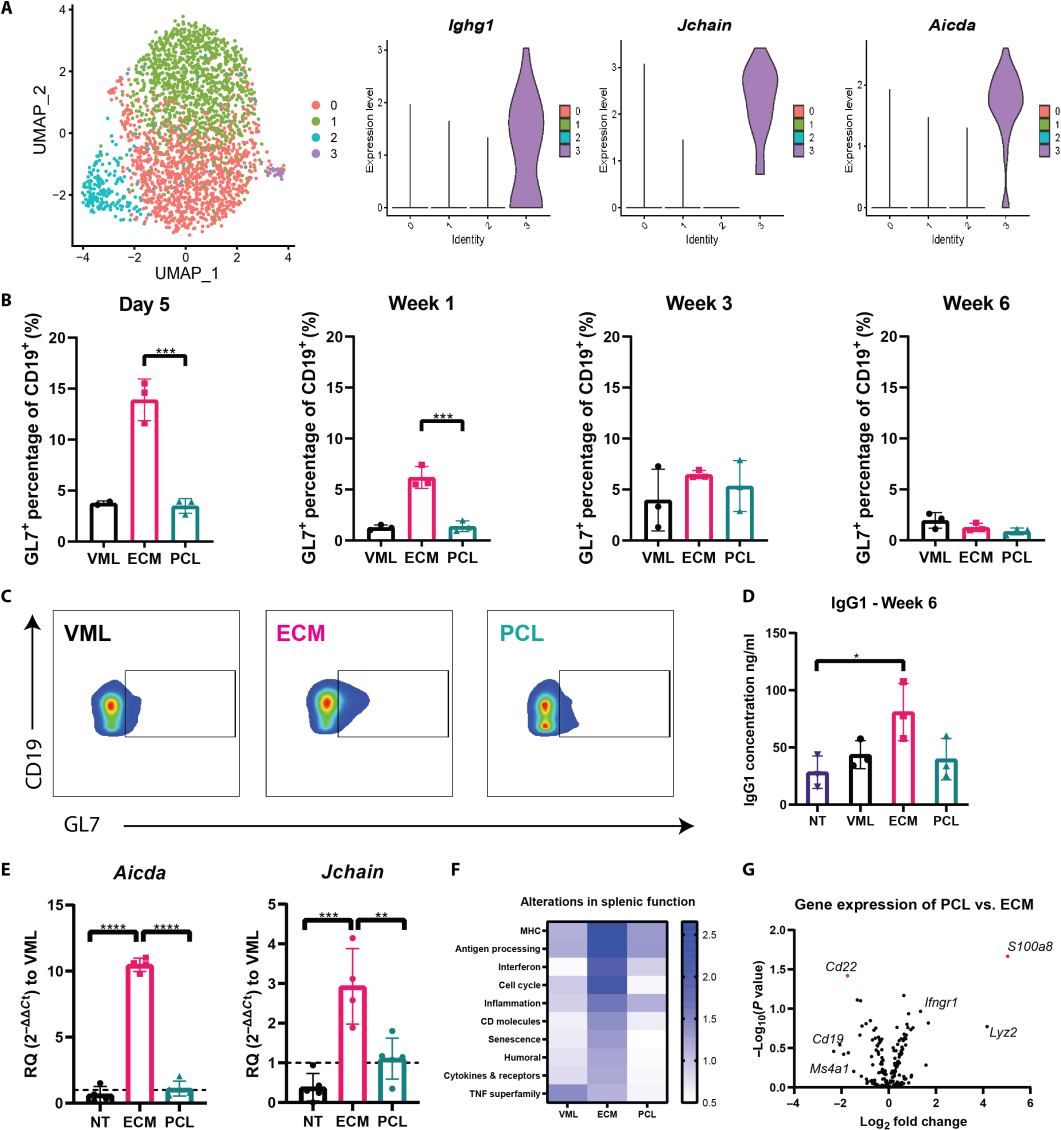
ECM induces germinal center formation. (**A**) Dimensional reduction projection of B cells onto two dimensions using UMAP. Cells are colored by cluster. Violin plots of cluster gene expression of surface markers identified by differential expression analysis demonstrating genes highly expressed in cluster 3 associated with germinal center formation. (**B**) Flow cytometry counts of GL7^+^ B cells (defined as GL7^+^CD19^+^) in the draining LN following injury ± biomaterial shown as bar graphs for each time point. (**C**) Flow cytometry plots of day 5 GL7 on B cells comparing VML, ECM, and PCL. (**D**) Gene expression using qRT-PCR at 1-week expression of *Aicda* and *Jchain* compared to VML. (**E**) Serum analysis of IgG1 at 6 weeks after injury. (**F**) Heatmap of NanoString pathway scores in which the scale reflects the *z* score of each pathway at 3 weeks after injury in the spleen. (**G**) Volcano plot of PCL to ECM gene expression differences, where *S1008a* and *Cd22* are divergently regulated. Data are means ± SD, *n* = 4, one-way ANOVA with subsequent multiple comparison testing. (C) ****P < 0.0001, ***P < 0.001, **P < 0.01, *P < 0.05. VML indicates injury with saline, ECM indicates injury with ECM, and PCL indicates injury with PCL.

Because the 3-week time point resulted in an increase in CD19^+^B220^+^ B cells in the ILN, we further characterize the B cell response to biomaterial scaffolds at week 3 following injury. We performed 10x 5′ single-cell RNA sequencing (RNA-seq) with transcriptome and B cell receptor (BCR) sequencing on CD19^+^B220^+^ B cells isolated from the ILN for all three groups: injury with saline, injury with SIS-ECM, and injury with PCL-treated mice. CD19^+^B220^+^ B cells and CD3^+^ T cells were isolated from the ILNs 3 weeks after each biomaterial implantation. After alignment with Cell Ranger, the counts were log-normalized using total library size, centered, and scaled before input to principal components analysis (PCA). The top 50 principal components were used for input to UMAP (uniform manifold approximation and projection) and clustering, which provide a visualization of the data and group the cells, respectively. UMAP differentiated cell populations based on B cell identifiers *Cd19* and *Ms4a1* (fig. S4A). Unbiased clustering algorithms categorized B cells into four clusters (Fig. 2A): two clusters in which the cells were largely undifferentiated (clusters 0 and 1), one cluster containing B cells with a type 1 interferon phenotype (cluster 2), and one cluster of B cells characterized by B cell maturation (cluster 3) (Fig. 2A). ECM treatment up-regulated genes associated with germinal center formation and class switching of B cells found in cluster 3 (Fig. 2A), including *Ighg1*, *Jchain*, and *Aicda*.

To further probe the single-cell cluster 3, we conducted multicolor flow cytometry, gene expression analysis, and immunoglobulin serum assessment. To validate the B cell maturation specific to the ECM condition found in the single-cell analysis, we probed expression of GL7 via flow cytometry (Fig. 2, B and C). GL7 is expressed in activated B cells, but not naïve B cells, and is frequently used as a marker of germinal centers in secondary lymphoid organs (*27*). GL7 increased in the ILN in response to ECM biomaterial implantation. ECM stimulated the greatest increase in GL7^+^CD19^+^B220^−^ B cells as a percentage of the total CD19^+^B220^−^ B cell population at day 5 after injury (Fig. 2B); GL7^+^CD19^+^B220^−^ B cells comprised 13.9 ± 2.0% of cells present in the ILN after treatment with ECM, compared to 3.5 ± 0.7 and 3.7 ± 0.2% in injuries treated with PCL and saline, respectively. Immunoglobulin serum analysis assessment further suggested differences in antibody production based on biomaterial treatment. ECM induced elevated levels of immunoglobulin G1 (IgG1) after 6 weeks, indicating that class switching had occurred in ECM-treated groups compared to non-injured/no-biomaterial (NT) groups (Fig. 2D). IgM increased in all groups 6 weeks after injury (fig. S4C). In addition, we probed expression of *Aicda* and *Jchain* in the ILN 1 week following injury with quantitative polymerase chain reaction (qPCR) and assessed that *Jchain* is expressed on all differentiated B cells and *Aicda* is expressed by B cells during germinal center development (*28*, *29*). ECM induced a 10-fold increase in *Aicda* expression compared to saline and PCL treatment groups (Fig. 2E) and a similar induction of increased expression of *Jchain* expression.

The single-cell sequencing of draining ILNs also provided insight into the divergent B cell responses to ECM versus PCL materials. To identify intercellular signaling patterns connected to transcription factor activation from single-cell data, we used the DOMINO analysis tool as described by Cherry *et al.* (*30*). Notably, B cells up-regulated *Icosl* expression in ECM-treated animals (fig. S4B), which is an important contributor to T follicular helper (T_FH_) cells and B cell interactions in germinal centers. T_FH_ cells aid in triggering and propagating the formation of germinal centers through interactions with B cells in the LN (*31*). The interactions between T_FH_ and B cells lead to the development of high-affinity bone marrow–differentiated B cells (*31*). This, along with the greater proportion of GL7^+^CD19^+^B220^−^ B cells at early time points (Fig. 2, B and C) and greater levels of class-switched IgG1 subclass antibodies (Fig. 2D), suggests that ECM biomaterials may promote high-affinity T cell–dependent B cell responses in the regenerative microenvironment. While these indicators of germinal center GL7^+^CD19^+^B220^−^ B cell responses were not found in PCL-treated animals, there were significantly more CD138^+^CD19^+^B220^−^ B cells in the muscle compared to injury with saline (fig. S4D). This increase in CD138^+^CD19^+^B220^+^ B cells in the quad tissue could represent short-lived differentiated B cells that develop outside of germinal centers (*32*). This possibility is supported by the production of IgM without an increase in IgG (fig. S4C).

Antigen-specific B cell activation occurs through the BCR, so the presence of BCR clones provides insight into potential antigen specificity of the B cell response to biomaterials. V(D)J library processing was conducted to investigate changes in immunoglobulin heavy-chain VH recombination and repertoires in the single-cell B cell evaluation. ECM treatment produced the most clones compared to other groups (fig. S5), suggesting that ECM stimulates CD19^+^B220^+^ B cells to undergo somatic hypermutation to create a diverse repertoire of clones. As ECM is a mixture of xenogeneic polysaccharides and proteins, the development of ECM-specific clones is expected (*33*, *34*). However, clonal development was also observed in the PCL-treated group and in injury with saline control animals to a less marked degree, suggesting the possible development of some self-antigen–specific B cell clones. Sterile inflammation during muscle injury releases danger-associated molecular patterns (DAMPs) that can contribute to overcoming B cell tolerance (*35*), and evidence suggests that IL-17–producing T cells may also contribute to this process (*36*). The extent and importance of self-directed B cell responses in the context of biomaterial-mediated wound repair is an intriguing area for future study. Overall, while further study is needed, the results here contribute to a developing view of the potential responses to natural and synthetic materials during wound repair that also include the types of induced B cell responses.

In tandem with our B cell sequencing, we conducted sequencing on isolated CD3^+^ T cells from the ILN. In line with our previous work, which provides evidence that antigen-specific T cell responses correlated with the presence of PCL implants (*20*), *Cd40lg* gene expression increased in T cells of PCL-treated animals via single-cell data (fig. S6). *Cd40lg* encodes *Cd40l* (also commonly referred to as *Cd154*), a costimulatory molecule up-regulated by activated T cells (*31*, *37*). *Lck,* the gene for a tyrosine kinase involved in signaling through the T cell receptor (TCR) complex, was also up-regulated in T cell clusters from PCL-treated mice. T cells from mice treated with either ECM or PCL showed up-regulation of genes related to adhesion and migration, such as *Sell* (which encodes *L-selectin/Cd62l*) and genes for integrin receptors. T regulatory cells (T_regs_) in PCL-treated animals showed up-regulated *Tnf* expression, which could be indicative of inflammatory, T_H_17-like T_regs_ that have been implicated in autoimmune and inflammatory disease pathogenesis (*38*).

To complement the injury site and ILN interrogation of B cells, we also conducted analysis on splenic B cells. As B cells in the spleen can provide information on the systemic response (*39*), we sorted CD19^+^B220^+^ B cells from the spleen and performed multiplex gene expression analysis 3 weeks after biomaterial implantation. Using NanoString nSolver software, pathway analysis was derived from each gene’s normalized expression and associated pathways in the NanoString Immune Profiling Panel (*40*). There was a notable difference in the gene expression profile of splenic B cells depending on biomaterial treatment. Splenic CD19^+^B220^+^ B cells from mice implanted with the ECM biomaterials up-regulated genes associated with antigen processing, MHC presentation, and pathways associated with cell cycle genes when compared to injury with saline and PCL groups (Fig. 2F). MHC genes up-regulated in ECM treatment group include *H2-K1*, *H2-Aa*, and *H2-T23* compared to B cells sorted from injury with saline (fig. S7, A and B). ECM treatment corresponded with up-regulated CD molecule presentation, humoral-associated genes, and cytokines/receptors (Fig. 2F). In contrast, splenic B cells from mice treated with PCL significantly up-regulated *S100a8*, an alarmin typically associated with environmental inflammation (*41*, *42*) compared to injury with saline (Fig. 2G and fig. S7).

### B cells increase antigen presentation and contribute to fibrosis with PCL

**Fig. 3.**
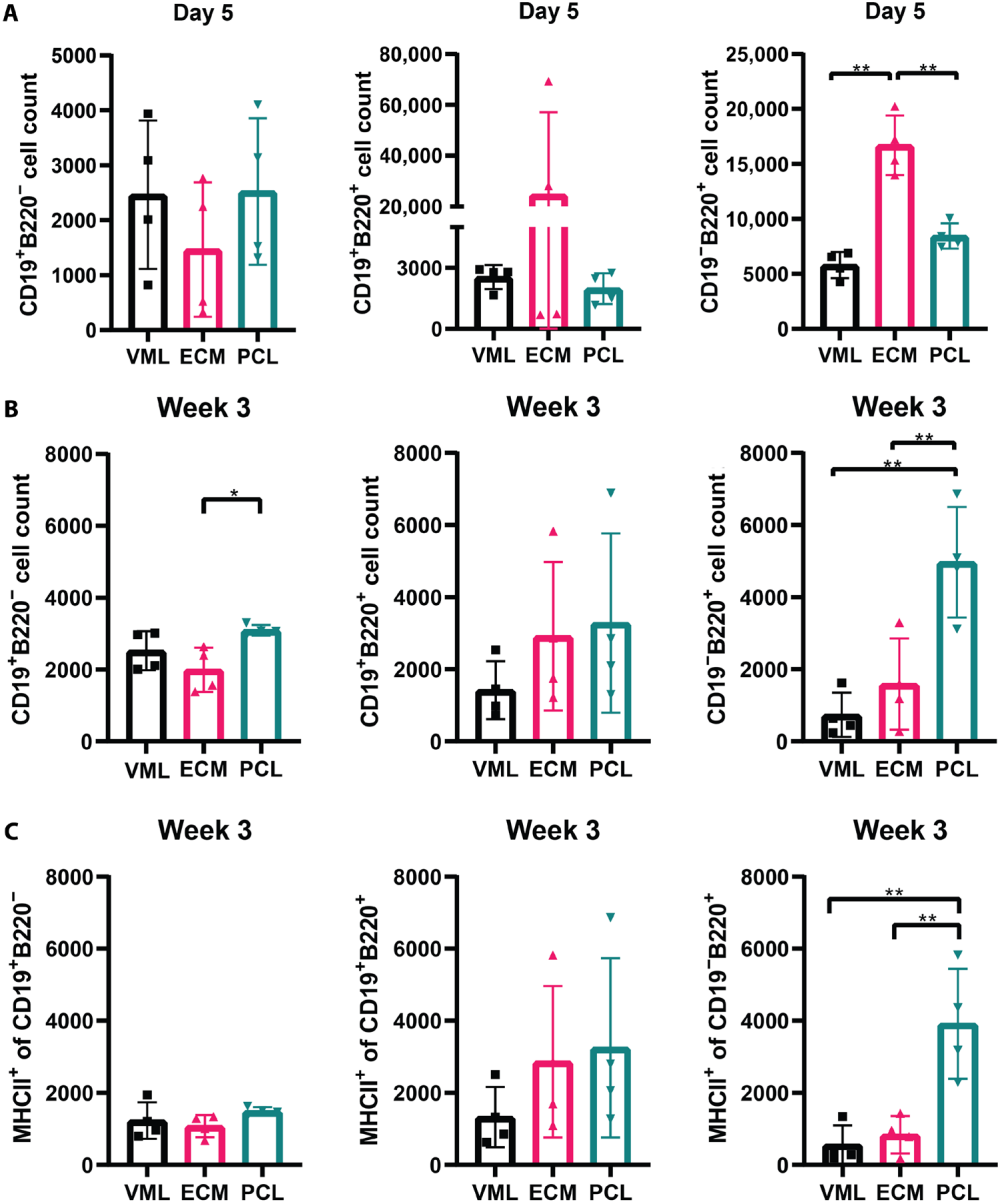
PCL induces B cell antigen presentation in the quad tissue. (**A**) Flow cytometry counts of B cell subsets: CD19^+^B220^−^, CD19^−^B220^+^, and CD19^+^B220^+^ in the quad tissue at day 5 after injury. (**B**) MHCII^+^B220^+^ cell population in quadricep tissue at day 5 after injury. (**C**) Flow cytometry counts of B cell subsets: CD19^+^B220^−^, CD19^−^B220^+^, and CD19^+^B220^+^ in the quad tissue at week 3 after injury. Data from CD19^−^B220^+^ are also used in Fig. 1A. Data are means ± SD, *n* = 4, two-way ANOVA with subsequent multiple comparison testing [(B and C): *P < 0.05, **P < 0.01]. VML indicates injury with saline, ECM indicates injury with ECM, and PCL indicates injury with PCL.

PCL implantation resulted in two peaks of CD19^−^B220^+^ B cells in the injured muscle tissue (day 5 and 3 weeks after implantation), while ECM implantation resulted in a peak of CD19^−^B220^+^ B cells at day 5 (Fig. 3A). The second peak in CD19^−^B220^+^ B cell number occurred following treatment with PCL at 3 weeks after implantation, while in the other treatment groups, B cell numbers decreased (Fig. 1A). At 3 weeks, CD19^−^B220^+^ and CD19^+^B220^+^ B cells increased in the tissue with both PCL and ECM implants compared to injury alone (Fig. 3B). However, the increase in CD19^−^B220^+^ B cells was significantly higher in the PCL group compared to the ECM at 3 weeks (Fig. 3B). Furthermore, PCL implantation resulted in significantly higher numbers of antigen-presenting MHCII^+^ B220^+^ cells in the tissue 3 weeks after implantation compared to the other conditions (Fig. 3C). PCL treatment further correlated with differentiated B cell phenotypes (CD138^+^) seen with increased recruitment of CD138^+^B220^+^ B cells compared to ECM (fig. S8).

**Fig. 4.**
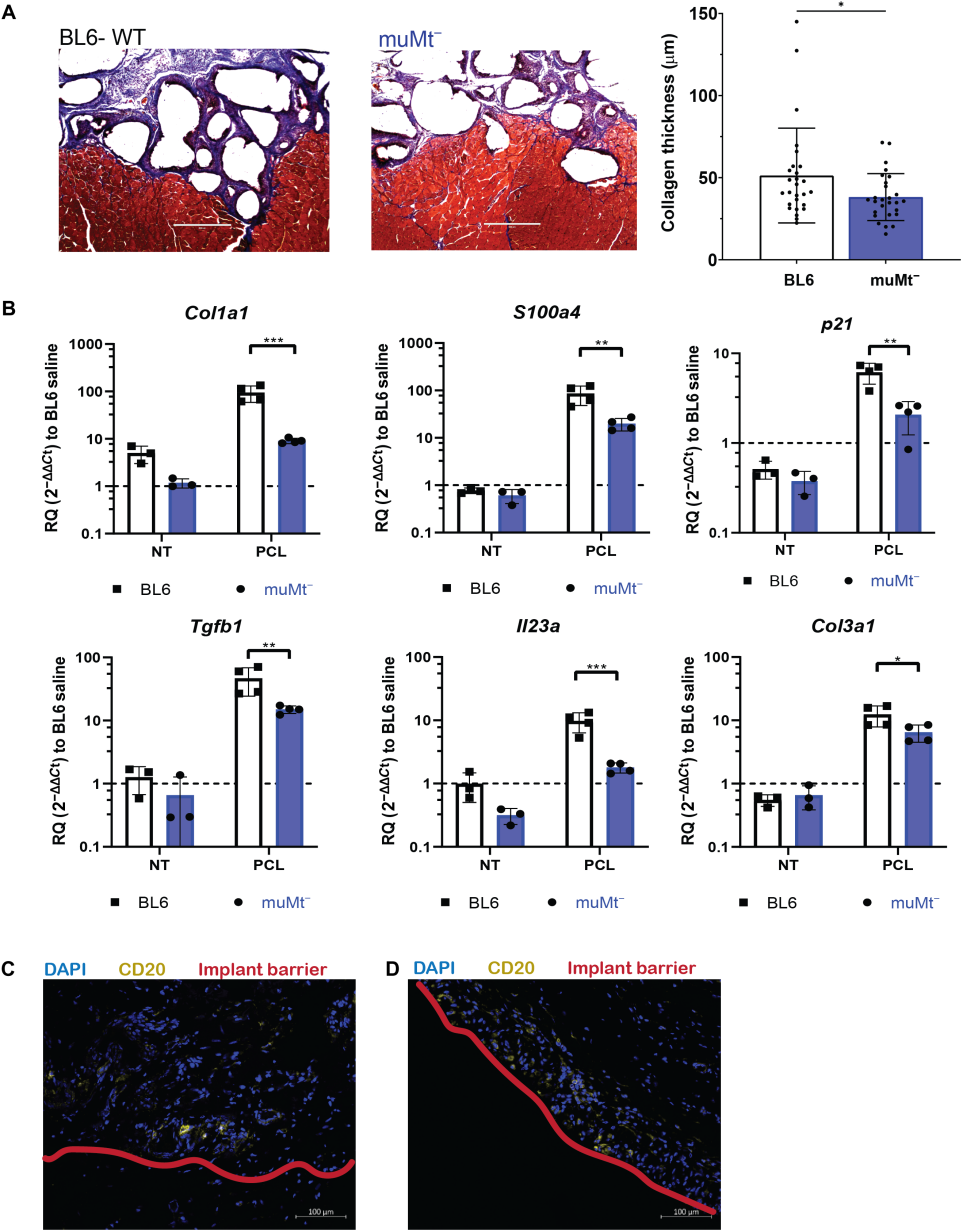
muMt^−^ reduces PCL-mediated fibrosis. (**A**) Histological staining of 6-week PCL implants in BL6-WT and muMt^−^ with Masson’s trichrome for collagen fibrils. Scale bar, 400 μm. (**B**) Gene expression analysis of fibrosis-related genes including *Col1a1*, *S100a4*, *p21*, *Tgfb1*, *Il23a*, *Col3a1* via qRT-PCR. (**C**) Immunofluorescence staining of CD20^+^ cells (yellow) and the implant barrier (red) in human tissue samples surrounding breast capsule implants of acellular adipose tissue (AAT) and (**D**) of silicone. Scale bar, 100 μm. Data are means ± SD, *n* = 3 for NT controls, *n* = 4 for PCL biomaterial, two-way ANOVA with subsequent multiple comparison testing [(C): ***P < 0.001, **P < 0.01, *P < 0.05]. muMt^−^ indicates mice that lack mature B cells, BL6 indicates C57BL6 mice, VML indicates injury with saline, ECM indicates injury with ECM, and PCL indicates injury with PCL.

To evaluate the functional impact of B cells in the FBR, we implanted the biomaterials in muMt^−^ mice, a homozygous mutant mouse strain that lacks mature B cells. In the muMt^−^ mice, there were no differences in the percentage of macrophages or phenotype (CD86^+^ and CD206^+^) compared to WT at 1 week (fig. S9A). No statistical differences were detected in numbers of CD4^+^ or CD8^+^ cells compared to WT at 1 week after injury with biomaterials ECM or PCL (fig. S9B). NK1.1 and γδ T cells decreased in the ECM biomaterial response at 1 week after injury when compared to the WT (fig. S9B). At 6 weeks after injury, there was no statistical difference in cytokines associated with the PCL profibrotic response (IL-17) in the muMt^−^ mice (fig. S9C). Histological assessment of fibrosis around the PCL implants confirmed the reduced fibrosis in the muMt^−^ mice. PCL in muMt^−^ tissue injury yielded reduced collagen matrix deposition via Masson’s trichrome compared to WT with PCL (Fig. 4A).

In addition, there were significant differences in genes associated with fibrosis and the FBR. In the muMt^−^ mice implanted with PCL, *S100a4*, *Col1a1*, *Col3a1*, and *Tgfb1* expression significantly decreased compared to WT mice implanted with PCL (Fig. 4B). *Il23* and *p21* also significantly decreased in the muMt^−^ mice (Fig. 4B).

Last, to determine potential clinical relevance, we evaluated CD20^+^ B cells in tissue samples from human breast implant tissue expanders, which have been previously demonstrated to exhibit an FBR and type 3 (IL-17–mediated response) (*4*, *20*). CD20^+^ is the human marker for B cells. Small clusters of CD20^+^ B cells (Fig. 4C) were present in tissue adjacent to the implant. We also assessed the B cell response in an ECM material, acellular adipose tissue (AAT). CD20^+^ B cells (Fig. 4D) were also present in the AAT and in the tissue next to the implant.

## DISCUSSION

The innate immune response to tissue damage and biomaterials is well recognized, along with a tissue specificity to this behavior. Tissue damage signals after injury activate multiple cell types through multiple pathways, including neutrophils, eosinophils, monocytes, and macrophages, among other immune cells. Neutrophils can orchestrate and prime adaptive immune cell responses through secretion of chemokines/cytokines and the release of neutrophil extracellular traps (*43*). Chronic neutrophilia is also associated with type 3 (IL-17 immune responses) (*44*). Eosinophils are of particular interest in the realm of natural biomaterials, as these cells can prime the local tissue response and stimulate an IL-4/type 2 immune response that can contribute to wound healing (*45*). Monocytes and macrophages are also known to alter their immune phenotypes along a wide spectrum of proinflammatory and proresolution outcomes in response to biomaterials (*46*, *47*). Their phenotypes are influenced by numerous factors, including the tissue-specific immune environment and biomaterial physical-chemical properties that direct tissue healing and repair (*48*). The paradigm of the FBR to biomaterials has traditionally focused on these innate immune cells and their impact on fibrosis. However, there is growing recognition of the adaptive immune response to tissue damage and repair. For example, Ramos and colleagues (*49*) found that antigen-specific T cells recognizing tissue-specific ECM molecules developed after cardiac injury. In the case of central nervous system injury, Kipnis and colleagues (*50*) found that MHCII was required for T_H_2-mediated repair. Tissue damage can occur with biomaterial implantation and tissue remodeling, suggesting that an adaptive immune response may also occur (*20*, *51*). T cell responses to both synthetic and naturally derived biomaterials have now been demonstrated in multiple studies (*20*, *51*).

B cells are known to participate in the immune response to tissue damage and wound healing through antibody production and cytokine secretion (*52*, *53*). Following injury, IgM and IgG antibodies rapidly increase in the tissue and blood, where they accelerate critical functions in healing by binding to wounded tissues, inducing phagocytosis by Fc receptors on neutrophils or macrophages (*52*, *53*). Autoantibodies are present in the injury site and connect both the local injury immune response and splenic responses, as splenectomies result in prolonged presence of neutrophils and macrophages in an injury site (*53*). Multiple studies have defined direct roles for B cells in repair of skin excisions and bone fractures along with repair of kidney damage and intestinal injury (*12*, *54*, *55*). The addition of B220^+^CD19^+^ B cells directly onto diabetic wound skin excisions substantially accelerated neutrophil invasion, decreased apoptosis in the wound bed, and reduced time to skin closure (*12*). Regulatory B10 cells, which secrete IL-10, mediate intestinal inflammation and orchestrate bone fracture repair in a time-sensitive manner (*54*).

B cell research in the context of medical implants historically focused on antibody production. In particular, the concerns over deleterious responses to breast implants in the 1990s prompted multiple safety studies (*56*). These studies of breast implant safety primarily investigated antibody production, specifically antibodies that might recognize the implant itself. They found that patients who had received silicone elastomer implants had elevated anti-polymer reactive antibodies in the serum, which were nonexistent in nonimplanted patients. Further studies have disputed this finding, suggesting that antibody reactivity was to the hydrophobic nature of the silicone and not implant specific (*57*). Review of the safety studies by the U.S. Institute of Medicine in 2000 led to an inconclusive finding, and silicone implant use has since continued in the clinic (*58*). While ongoing work seeks to assess the presence and antigen specificity of antibodies, B cells in wound healing are not limited to antibody production. The functions of B cells, the generation of germinal centers in response to wounds, and the response of the splenic B cells can also highlight additional B cell contributions to wound healing and to the biomaterial response.

Biomaterial scaffolds have previously been shown to induce B cells to distinct phenotypes and functions. Polymeric nanoparticles and vaccines serve as both a delivery vehicle and an adjuvant to enhance recognition and antibody production. Babensee and Sefton (*59*) found that polymeric scaffolds serve a deleterious adjuvant function that enhances recognition of transplanted cells that decreases cell viability and regenerative function. In this work, we investigated the B cell response to a biological ECM scaffold and a synthetic polyester. While this work demonstrates the diverging response of one natural and one synthetic biomaterial, other research noted the role of size, shape, and composition on the immune response and on the FBR (*60*, *61*). For example, alginate materials demonstrated B cell responses that were important in propagating fibrosis (*13*). In addition, previous work in our laboratory has commented on the B cell and antibody responses to collagen, poly(ethylene glycol), other tissue derivatives of ECM, and silicone (*20*, *51*). In this case, ECM-based materials contain proteins that serve as obvious antigens to an adaptive material response (*62*). Furthermore, Allman and colleagues (*62*) presented systemic T_H_2 activity with ECM exposure, confirming that both arms of the adaptive immune system are activated with these materials. These studies support our results that show B cell development following treatment with SIS-ECM material. Proteomic analysis of biologic scaffolds demonstrates a complex mixture of collagen, ECM glycoprotein, and even intracellular proteins that could be candidate antigens (*34*, *63*). Mice treated with ECM after injury demonstrated elevated levels of IgG1 and IgM at 6 weeks, which has previously been demonstrated to facilitate wound healing in a clinical model of skin regeneration (*53*). This introduces a previously unknown perspective of B cell response to biomaterials, promoting the classification of both the antibodies and B cell subsets found in wounds. Lack of B cells did not appear to affect the response to ECM, although tissue repair was not studied in detail.

In contrast to ECM-based biomaterials, there is no obvious source of antigens in the synthetic biomaterial. Determining the direct antigen is a challenging endeavor but could be related to including tissue damage–related self-antigens, adsorbed proteins, and non–peptide-repeating structures (*20*, *64*). Results from this study highlight that PCL implantation increases antigen presentation by B cells. B cells have a unique capacity for antigen presentation that has been implicated in infection, disease pathogenesis, and autoimmunity (*65*–*67*). Specifically, B cells present specific antigens to activate other B cells and cognate T cells. The specific presentation of antigens can induce T cell proliferation and has been seen to promote a type 2 wound healing response in chronic allergic lung diseases (*65*). B cells are also capable of nonspecific antigen presentation, which results in inactivation of T cells or induction of regulatory T cell differentiation (*68*). The efficacy and efficiency through which B cells serve as antigen-presenting cells are tightly regulated, as MHCII presentation is dependent on B cell developmental stage. As with all things in immunology, the exact role of B cells as antigen-presenting cells is context dependent. However, it is known that B cell antigen presentation is a highly coordinated uptake, which induces cell polarization and usually occurs in the secondary lymph organ.

Splenic B cells also expressed genes associated with inflammation in response to PCL implantation, such as *S100a8,* suggesting that B cells are participating in the FBR beyond antigen presentation. Previous work demonstrated that the connection between senescent stromal cells, fibroblast, and IL-17 production from immune cells is implicated in fibrosis (*20*). The changes in *p21* and *Il23* suggest that B cells contribute to the IL-17 senescence axis. Lack of mature B cells in the muMt^−^ mice appeared to reduce fibrosis around the materials, further supporting the role of B cells in the FBR fibrotic response. While we only studied two materials here, we provide a detailed analysis into the spectrum of possible B cell responses to biomaterials. They not only were selected as examples of different compositions but also produced distinct immune responses: a type 2 or 3 (*17*) immune response. Other materials will likely produce responses that fall somewhere in the middle on this wide spectrum of material properties and responses.

Traditionally, the FBR to biomaterial implants and scaffolds has been considered primarily a local phenomenon. The involvement of adaptive immune cells, such as T and B cells, in the FBR and tissue repair process opens the door to considering a systemic immune response. Here, we found changes in B cell expression profiles in the local draining LNs and the spleen. Gene expression patterns in the splenic B cells shifted with injury alone and further differentiated depending on the biomaterial composition. While B cells may be small at the local injury or implant site, they have physiological impact, particularly in the case of synthetic materials. The potential interplay between a local implant and systemic factors introduces the concepts that a biomaterial may affect the overall physiological state of an organism, and vice versa, the physiological state of the organism may affect the response to injury, implants, and scaffold repair strategies.

## MATERIALS AND METHODS

### Surgical injury and material implantation

All animal procedures were performed with approval by Johns Hopkins University Institutional Animal Care and Use Committee. The animals were all female mice aged 6 to 9 weeks old. Two strains were used: WT C57BL/6J (stock no. 00064, The Jackson Laboratory) and muMt^−^ (mature B cell KO) on C57BL/6J background (stock no. 002288, The Jackson Laboratory). Bilateral traumatic muscle defects were created as previously described (*20*, *51*, *69*). While under anesthesia, a 3 mm by 3 mm by 4 mm section of quadriceps femoris tissue was excised from both hindlimbs. Following injury, the quadriceps muscle defects were filled directly with 30 mg of a synthetic (PCL, particulate, *M*_n_ = 50,000 g/mol; mean particle size <600 μm; Polysciences) or biological (ECM, decellularized porcine small intestine submucosa) scaffold material. For control surgeries (considered no-implant with injury controls), 0.05 ml of phosphate-buffered saline was dispensed directly into the wound. All materials were disinfected with ultraviolet before use. Immediately after surgery, mice were given subcutaneous carprofen (5 mg/kg; Rimadyl, Zoetis) for pain relief. Mice were euthanized for sample harvests at days −3 and 5, days 1 and 3, and 6 weeks after surgery.

### Sample collection and digestion for single-cell analyses

Murine tissue samples (quadriceps femoris muscle and ILNs) were obtained for single-cell flow cytometry and/or fluorescence-activated cell sorting (FACS). Quad tissue samples were finely diced and digested with Liberase TL (1.67 Wünsch units/ml) (Roche Diagnostics) and deoxyribonuclease I (0.2 mg/ml; Roche Diagnostics) in RPMI 1640 medium (Gibco) for 45 min at 37°C. The digested quad tissues were ground through 70-μm cell strainers (Thermo Fisher Scientific) and rinsed with RPMI 1640 + 5% fetal bovine serum. Percoll (GE Healthcare) density gradient centrifugation was used to remove debris from quad digestions and enrich the leukocyte fraction. ILN samples were ground through 70-μm cell strainers with excess RPMI 1640 + 10 mM Hepes buffer solution. The enriched single-cell suspensions were washed and stained following antibody panels (tables S1 to S3), with respect to the intended application. No differences in single-cell isolation from different material environments were observed.

### qRT-PCR assay

Isolated tissues were digested using TRIzol reagent to isolate total RNA. RNeasy Mini Kit (Qiagen) purified mRNA from total RNA. Quantitative reverse transcription PCR (qRT-PCR) was performed using TaqMan Gene Expression Master Mix (Applied Biosystems) consistent with the manufacturer’s instructions. In brief, 2 μg of total RNA was used to synthesize complementary DNA (cDNA) using SuperScript IV VILO Master Mix (Thermo Fisher Scientific). The cDNA concentration was set, according to the manufacturer’s recommendation, at 100 ng per well (in a total PCR volume of 20 μl). qRT-PCR assays were performed as TaqMan single-plex assays on the StepOne Plus Real-Time PCR System (Thermo Fisher Scientific), using the manufacturer’s recommended settings for quantitative and relative expression. For tissue samples, *Hprt*, *Rer1*, *Oaz1* were used as the reference gene, and samples were normalized to either no-treatment or injury with saline depending on application. All qRT-PCR data were analyzed using the Livak method, where ΔΔ*C*_t_ values are reported as relative quantification (RQ) values calculated by 2^−ΔΔ*C*t^. RQ values are represented by arithmetic means, with arithmetic SD represented by error bars. Low-expressing mRNA transcripts were preamplified using 250 ng of cDNA and the TaqMan Pre-Amp System (Thermo Fisher Scientific) with 14 cycles of amplification according to the manufacturer’s recommendation. Primer probes used for qRT-PCR and preamplification are listed in table S4.

### Flow cytometry and FACS

For cell isolation using FACS, the single-cell suspensions from digested quad and ILN samples were stained for 30 min at 4°C using Viability Dye eFluor 780 (eBioscience). For intracellular staining, the cells were stimulated with Cell Stimulation Cocktail (plus protein transport inhibitors) (eBioscience) diluted in complete culture medium (Iscove’s modified Dulbecco’s medium) supplemented with 5% fetal bovine serum for 4 hours. Cells were then washed, surface-stained, fixed/permeabilized with Cytofix/Cytoperm (BD), and then stained for intracellular markers. Flow cytometry was conducted using Attune NxT Flow Cytometer (Thermo Fisher Scientific). Antibody panels are listed in tables S1 to S3.

### NanoString gene expression analysis

B cells were isolated from ILN samples as previously described and stained for CD19 and CD3 (table S3). CD19^+^ cells were sorted using FACS, and mRNA was then isolated. After lysis in RLT Plus buffer containing 2-mercaptoethanol, the RNA was purified using the RNeasy Plus Micro Kit (Qiagen) and then quantified using High Sensitivity RNA ScreenTape (Agilent Technologies). RNA quality was assessed via 28S/18S area ratio, where only ratios ≥2.0 were used after being obtained via TapeStation Analysis software. The gene expression analysis for *n* = 3 biologically independent samples was conducted with the nCounter PanCancer Mouse Immune Profiling Panel (XT-CSO-MIP1-12, NanoString Technologies). On the basis of mRNA quantification, 50 ng of mRNA per sample was added to a barcoded probe set reagent and hybridized for 20 hours at 65°C according to the manufacturer’s protocol. NanoString data were processed with the nSolver 4.0 software kit according to the manufacturer’s protocol. Differentially expressed genes (*P* < 0.05 and positive fold change) for each sample group were used for further analysis. Differential gene expression analyses of each gene were performed using nSolver NanoString software. Pathway analysis was conducted from the annotated gene set global significance score (calculated as the square root of the mean squared *t* statistics of genes, informed by the gene expression analysis). Pathway score was used to summarize these results into a single score.

### Single-cell RNA-seq

The 10x Chromium instrument with 5′ v2 chemistry was used to generate single-cell RNA-seq libraries. Directly before loading in the chip, hashed samples were pooled in equal amounts. Encapsulation and library preparation were performed according to the manufacturer’s recommendations. Briefly, cells and beads are encapsulated in water droplets in a water-in-oil emulsion. Reverse transcription was then completed, tagging cell RNA and hashing antibodies with bead-specific oligonucleotide barcodes. We then ran whole-transcriptome amplification and separated the hashing antibody cDNA from the transcript cDNA. A sequencing library was generated for each portion. Last, we preferentially amplified BCR from an aliquot of the transcript cDNA and prepared a separate library for VDJ sequencing. Transcript and hashing libraries were pooled at a ratio of 9:1. The pooled libraries were sequenced to a depth of 50,000 reads per cell on the transcript library. The VDJ libraries were sequenced at 100,000 molecule reads per cell. In both cases, 10× recommended sequencing configurations were followed. To determine intra- and intercellular signaling occurring between the numerous cell subsets, we used DOMINO (github.com/chris-cherry/domino), a computational tool that allows identification of condition-specific intercellular signaling patterns connected to transcription factor activation from single-cell data (*30*).

### Transcriptome read alignment and processing

Read alignment for all three libraries was performed using Cell Ranger 3.0 recommended settings and the provided mm10 references where applicable. Seurat was used for processing steps where other software packages are not specified. Genes expressed in fewer than 0.1% of cells were removed, and cells with less than 500 unique molecular identifiers (UMIs) were removed. HTODemux was used with the hashing data to group cells into condition and remove condition doublets. Subsequent counts were log-normalized. Cell cycle scores were calculated using Seurat’s CellCycleScoring with previously defined gene sets (*70*), and the total percent of mitochondrial genes was calculated. Normalized gene expression values were scaled with linear regression of genes on cell cycle score, total UMI count, and percent mitochondrial gene contribution.

### PCA, dimensional reduction, clustering, and differential expression

PCA was performed on the scaled data, and the top 50 were used for downstream analysis. UMAP was used for dimensional reduction and visualization. A shared nearest neighbor graph was constructed from the principal components, and subsequent Louvain clustering was used to identify clusters. Mann-Whitney *U* tests were used to test differential expression of each cluster in comparison to all other cells in the dataset. The top 10 genes by fold change by cluster were used to generate a heatmap of differentially expressed genes.

### VDJ library processing

VDJ libraries were processed with Cell Ranger 3.0’s VDJ function with the provided mm10 references. Cell Ranger’s clonotype barcodes were then cross-referenced with hashing barcodes to label each cell for condition and singlet status. The resulting clonotype tables are reported (fig. S5).

### Serum/plasma (ELISAs)

Blood serum samples were collected by submandibular bleeding and diluted 1:20,000 using Abcam mouse IgG1, IgM, and IgG2a enzyme-linked immunosorbent assay (ELISA) kits (catalog nos. ab133045, ab133047, and ab133046, respectively). ELISA was performed according to the manufacturer’s recommended protocol. Briefly, the PCL-coated plate was blocked with the assay diluent for 1 hour before loading. Each serum dilution was loaded into the plate after blocking and incubated for 2 hours. After washing, anti-mouse IgG1, IgM, or IgG2a was added to conjugate the bound antibody for 1 hour. After washing, 30-min incubation with provided substrate solution, and addition of provided stop solution, absorbance was read at 450 nm with correction between 570 and 590 nm.

### Mouse samples for histopathology

Upon tissue harvest, immediate fixation was performed in 10% neutral buffered formation (48 hours), followed by stepwise dehydration to ethanol (200 proof). Tissues were then cleared with xylenes and paraffin-embedded. Sectioning of samples was performed with a Leica RM2255 microtome in a transverse orientation (7 μm thickness). For the LN staining, slides were deparaffinized and stained with Harris’s hematoxylin solution (Sigma-Aldrich, HHS32) for 3 min. Slides were rinsed with deionized water and immersed in tap water for 5 min. Next, slides were dipped three times in 0.33% acid ethanol until the background was clear. In the bluing step, slides were soaked in Scott’s tap water (Sigma-Aldrich, S5134) for 1 min and then rinsed with tap water three times. Slides were immersed in eosin (Sigma-Aldrich, HT110332) for 30 s, followed by dehydration and mounting with Permount mounting medium (Thermo Fisher Scientific, SP15). Imaging of the histological samples was performed on a Zeiss AxioObserver.Z2 microscope and processed using Zeiss Zen Blue software. The histopathology of the muscle tissue in the muMt^−^ mice was conducted with Masson’s trichrome (Sigma-Aldrich) according to the manufacturer’s protocols. Bright-field imaging of stained slides was performed. Collagen thickness was quantified between PCL particles from bright-field images of histological slices using ImageJ software (three slices per animal, average thickness of three images per slice).

### Human samples for histology

Tissue was acquired from patients undergoing breast implant exchange or replacement surgeries (for silicone implants, Johns Hopkins University Institutional Review Board exemption IRB00088842; for AAT, phase 1 clinical study was conducted at the Johns Hopkins University School of Medicine with IRB00027657). The silicone breast implants were surgical discards that were deidentified. Each section was weighed, and 0.25 g of tissue was dissected for histology. Peri-implant samples included tissues surrounding implants with both smooth and textured surface properties. Synthetic implants with a silicone shell were either temporary tissue expanders filled with saline or air or permanent implants filled with silicone or saline. All patients were female. Synthetic capsule samples were taken from patients with an average age of 56 years old (range of 41 to 70 years old). The synthetic capsule implantation time range spans 1 to 360 months. Alternatively, breast capsule implants with AAT were also assessed to contrast the synthetic implants. AAT patients’ average age was 48 years, and the average implant residence time was 32.5 days (*71*). All samples were prepared and stained in the following manner.

Tissues were harvested and fixed in 10% neutral buffered formalin for 24 hours before dehydration in EtOH, cleared with xylene, and embedded in paraffin. Samples were sectioned as 7-μm slices using the Leica RM2255 microtome. Samples were baked, deparaffinized, and refixed in neutral buffered formalin. Antigen retrieval was achieved by heating slides in 10 mM sodium citrate. Endogenous peroxidases were quenched using 3% H_2_O_2_ (Sigma-Aldrich), and aldehydes were quenched in glycine. Samples were incubated with 10% bovine serum albumin (BSA) (Sigma-Aldrich) and 0.05% Tween solution. Samples were incubated with anti-CD20 antibody (Abcam, ab78237) at 1:500 dilution for 10 min in 10% BSA. Samples were washed and underwent Opal HRP polymer incubation (Akoya Biosciences, ARH1000EA) for 10 min. Samples were washed again and underwent incubation with Opal 570 fluorophore in 1× amplification diluent (Akoya Biosciences) for 10 min. Samples underwent final washing and staining with 4′,6-diamidino-2-phenylindole (DAPI) stain for 5 min. Coverslips were applied, and slides were imaged using Zeiss Axio Observer with Apotome.2 and Zeiss Zen Blue software version 2.5.

## Notes

View/request a protocol for this paper from *Bio-protocol*.
