# Supplementary material for: Biomaterials direct functional B cell response in a material-specific manner
Source: Sci Adv. 2021 Dec 1;7(49):eabj5830. doi: 10.1126/sciadv.abj5830 (PMC8635437; doi:10.1126/sciadv.abj5830)
Supplement: Supplementary file 1 — Figs. S1 to S9 Tables S1 to S4 [file sciadv.abj5830_sm.pdf]

Science Advances
AAAS

Supplementary Materials for

Biomaterials direct functional B cell response in a material-specific manner

Erika M. Moore*, David R. Maestas Jr., Chris C. Cherry, Jordan A. Garcia, Hannah Y. Comeau, Locke Davenport Huyer, Sean H. Kelly, Alexis N. Peña, Richard L. Blosser, Gedge D. Rosson, Jennifer H. Elisseeff*

*Corresponding author. Email: jhe@jhu.edu (J.H.E.); moore.erika@ufl.edu (E.M.M.)

Published 1 December 2021, *Sci. Adv.* **7**, eabj5830 (2021)
DOI: 10.1126/sciadv.abj5830

The PDF file includes:

Figs. S1 to S9
Tables S1 to S4

Other Supplementary Material for this manuscript includes the following:

Data file S1

Supplementary Materials

(Data File IN ATTACHMENT)

Data files S1. Multiplex gene expression analysis of B cells sorted from the ILN 1 week after injury.

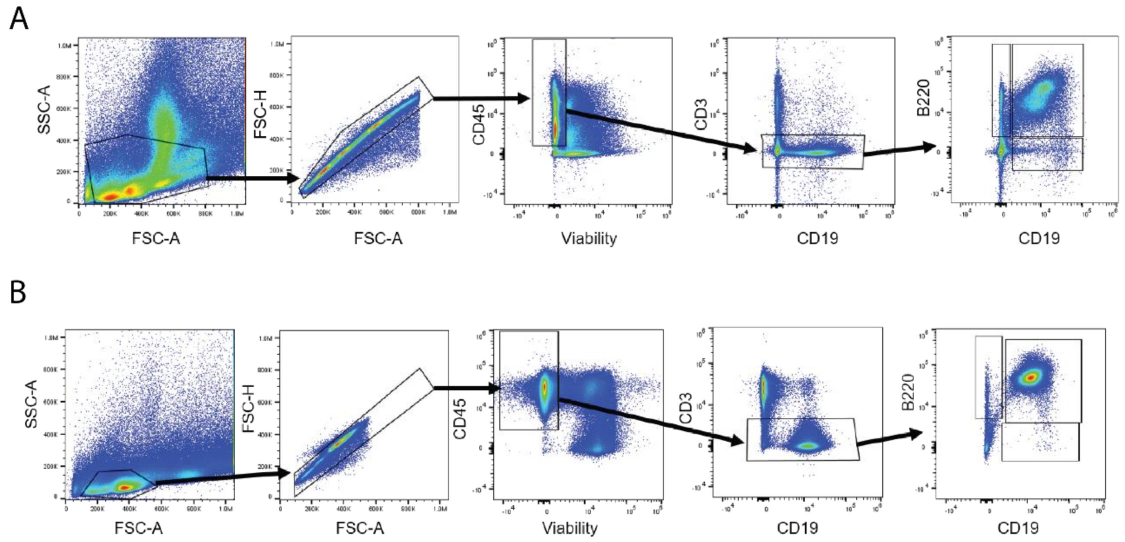

Fig S1. B cell flow cytometry gating strategy for **(A)** quadricep tissue and **(B)** draining lymph nodes when stained for viability, CD45, CD19, CD3, B220 and additional markers listed in Table S1 (accompanying analysis in Fig. 1,2, and 3). Additional gating steps are included when analyzing B cells for GL7, MHCII, and subsequent markers to characterize the B cell response more effectively.

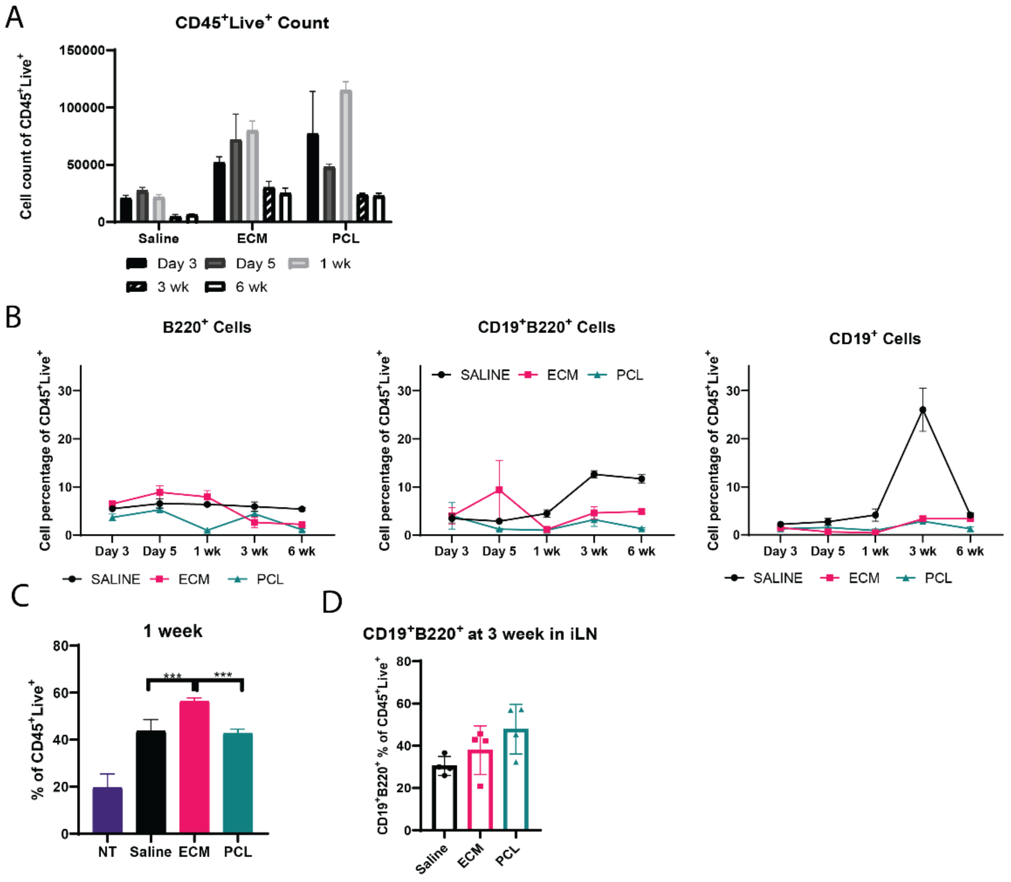

Fig. S2. Total immune and B cell content in quad tissue and draining inguinal lymph node after injury with or without biomaterial implant. (**A**) CD45$^+$ immune cells present in injured quad tissue over time. (**B**) Evolution of CD19$^+$B220$^-$, CD19$^+$B220$^+$, CD19$^-$B220$^+$ B cells as a percentage of the CD45$^+$ cells in the quad over time. (**C**) B220$^+$CD19$^+$ B cells in ILN 1-week post injury. **(D)** B220$^+$CD19$^+$ B cells in ILN 3 week post injury. Data are mean ± SD, n=4, Two-way ANOVA with subsequent multiple comparison testing [C: ** $p<0.001$, ** $p<0.01$, *$p<0.05$].

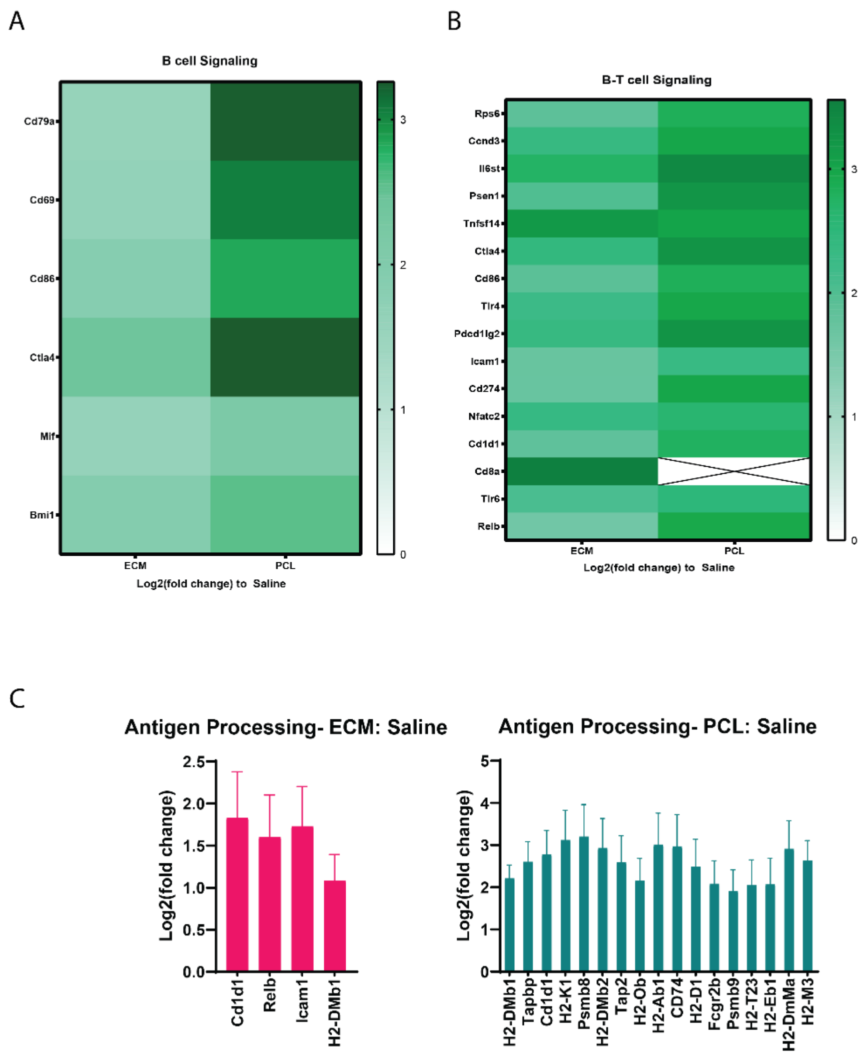

Fig. S3. Gene Expression related to (**A**) B cell signaling, (**B**) B-T cell interactions and (**C**) antigen processing isolated from $CD19^{+}B220^{+}$ B cells in iLN at 1 week. All treatment groups compared to VML injury with saline.

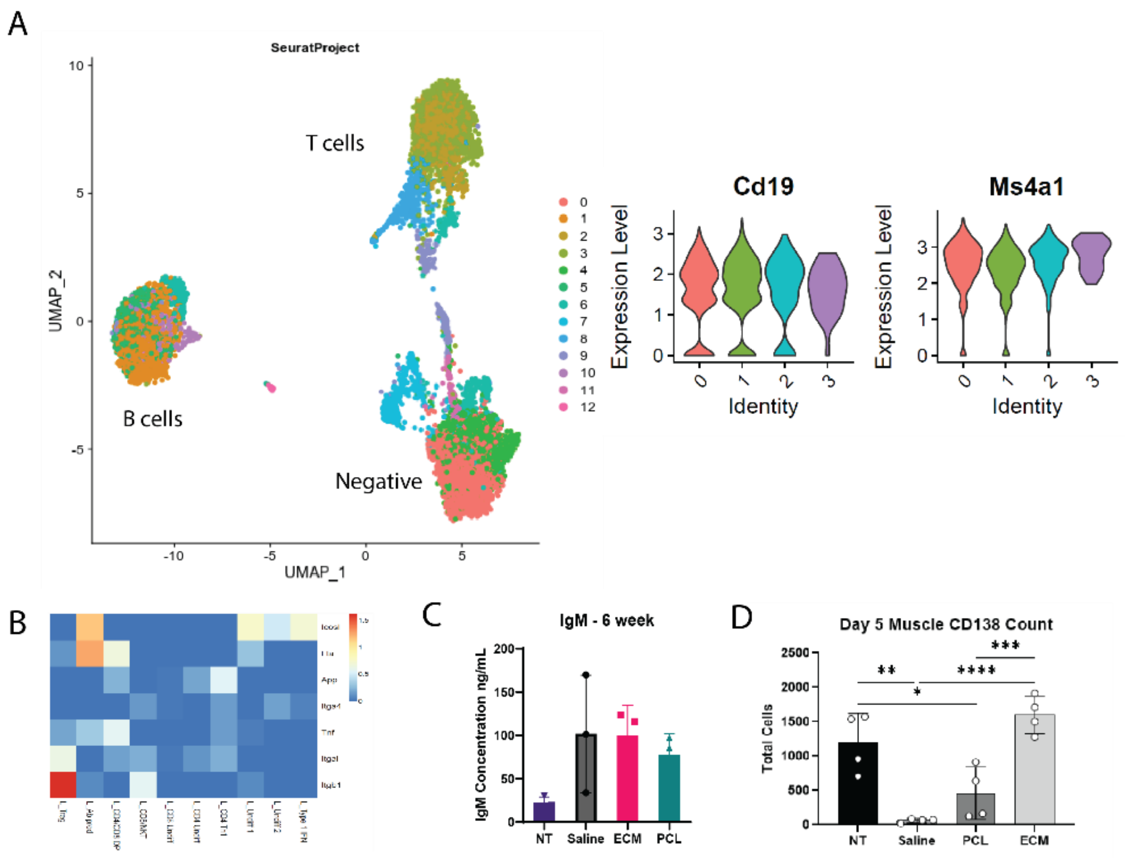

Fig. S4. Identification of B cell local and systemic response. (A) A uniform manifold approximation and projection (UMAP) of sorted B and T cells from ILN at 3 weeks. Differentiation of cells based on CD19 and Ms4a1. **(B)** T and B cell gene associations based on clustering in Part A. **(C)** IgM serum levels increased in each condition at 6 weeks post injury. **(D)** CD138+CD19+B220+ B cells in the quad tissue at Day 5 post injury. Data are means ± SD, n=4 for quad tissue, ANOVA [C: ** $p<0.001$, ** $p<0.01$, *$p<0.05$].

Cell Barcode	cond	IGK	IGH	IGL
**AGAGCTTAGCTGCAAG**	ECM	CLQYDEFPWTF		
**CATTCGCCAAGTACCT**	ECM	CLQYDEFPWTF		
**TTAACTCTCTAACTTC**	ECM	CLQYASSPYTF		
**ATGAGGGAGCAGCCTC**	ECM	CWQGTHFPQTF	None	
**CGAGCCATCAACGCTA**	ECM	CWQGTHFPQTF	CARDDYDERFAYW	
**CAGTCCTTCCTGCCAT**	ECM	CQQSNEDPYTF	None	None
**TACCTTATCATATCGG**	ECM		None	CALWYSNHWVF
**TGGCTGGTCTTTCCTC**	ECM	None	None	None
**ATCACGAGTTACCAGT**	ECM	CLQSDNLPLTF	CASHYYGSSYYFDYW	
**TGGGAAGCATGCAATC**	ECM	CLQSDNLPYTF	None	
**CTCGAAATCAGTGCAT**	ECM	CQQYNSYPYTF	None	None
**CCAGCGAAGAGGGCTT**	ECM	CFQGSHVPFTF	CARDDFDGVDYW	None
**TCGGGACCAACTTGAC**	ECM	CQQSNSWPYTF		
**TACGGGCCACGTAAGG**	Naive	CLQYASSPYTF	None	
**GAATGAAAGGCTCATT**	Naive			None
**ATAAGAGCAAGGTGTG**	Naive	CQQNNEDPYTF	None	
**GCACTCTAGGCAGTCA**	Naive	CQQGNTLPYTF	None	
**GACGCGTAGCCCTAAT**	Naive	CQQDYSSPFTF		None
**TCTGAGACAAATCCGT**	PCL	CHQWSSYPYTF	None	CALWYSNHWVF
**GTGGGTCCAGCCTATA**	PCL	CQQDYSSPYTF		
**AGTCTTTTCGCGTTTC**	PCL		None	CALWYSNHWVF
**AGCGTATCAGTATGCT**	PCL	CQQSNEDPYTF		
**AGGTCATTCGCCTGAG**	PCL	CQQNNEDPYTF	None	
**TACACGAGTAGCTGCC**	PCL	None	CARGGMEALYYYGREYFDVW	
**TGTATTCCACTCTGTC**	PCL		None	None
**CTGTTTAAGCTGCCCA**	PCL	CQQYYSYPYTF	None	
**GGATGTTAGAGATGAG**	PCL	CAQNLELPLTF	None	
**GGCCGATGTGCTAGCC**	Saline	CLQYDEFPWTF	None	
**GTGCTTCTCCGAAGAG**	Saline	CSQSTHVPWTF		
**GTCCTCAGTACATCCA**	Saline	CLQSDNLPLTF		
**CGAATGTCACGGTAGA**	Saline	CQHHYGTPFTF	CARDDYDNYAMDYW	
**CTCGGGAAGAGATGAG**	Saline	CWQGTHFPQTF		
**AGAGCGACACATCCAA**	Saline		None	CGVGDTIKEQFVYVF
**CACAAACTCATAACCG**	Saline	CQQYSSYPLTF		None

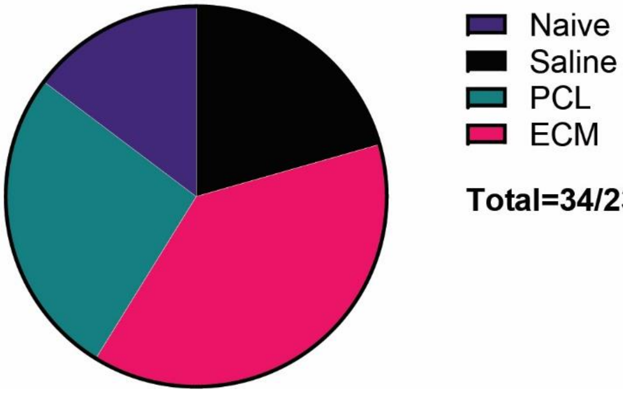

Total=34/2388 cells total

Fig. S5. VH Repertoires identified from single cell RNA sequencing. Clonal cells are shown by condition and clonal sequence. Pie plot shows number of clonal cells by condition.

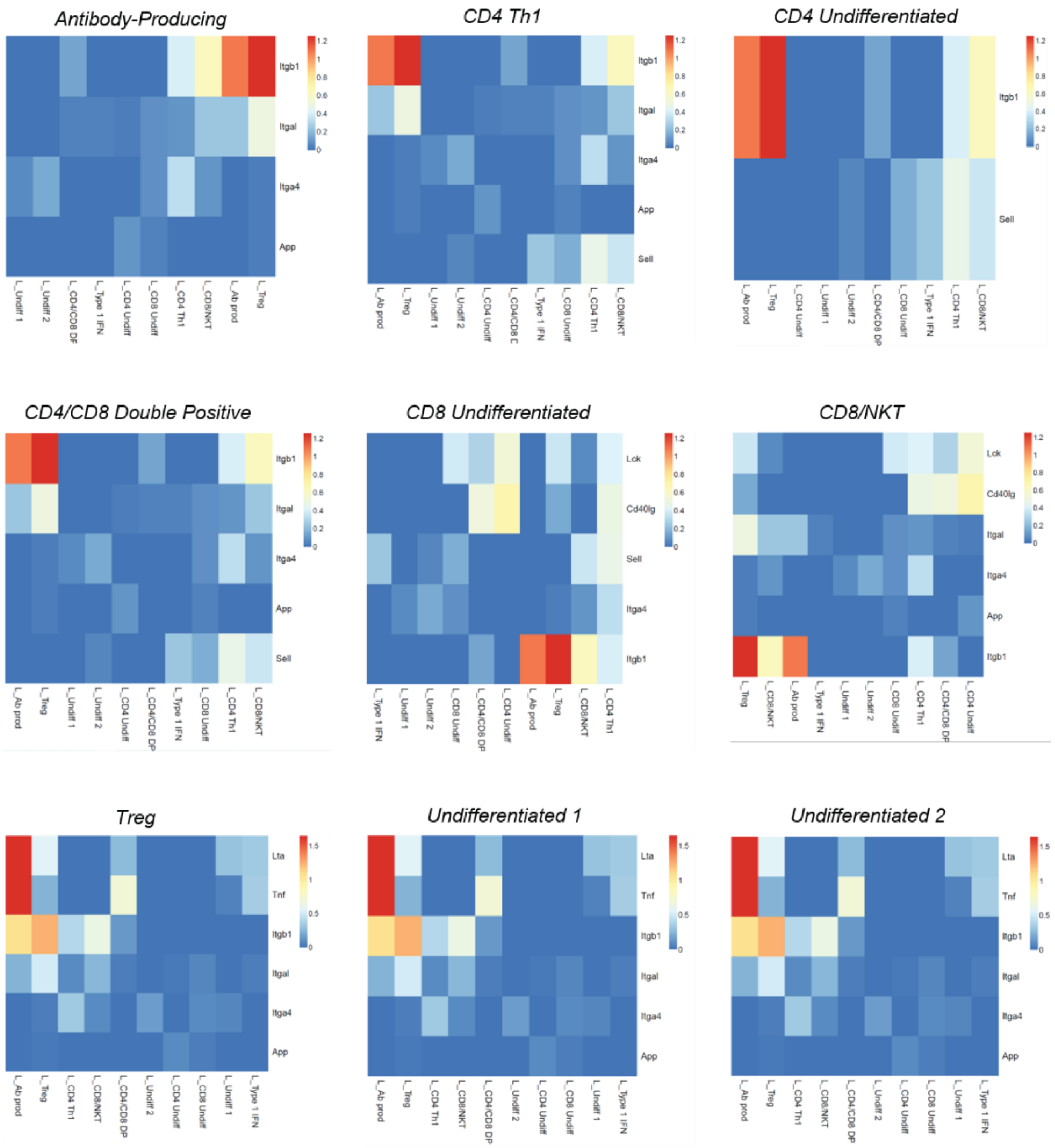

Fig. S6. Identifying B cell - T cell responses in PCL via single cell sequencing of each identified T cell and B cell cluster.

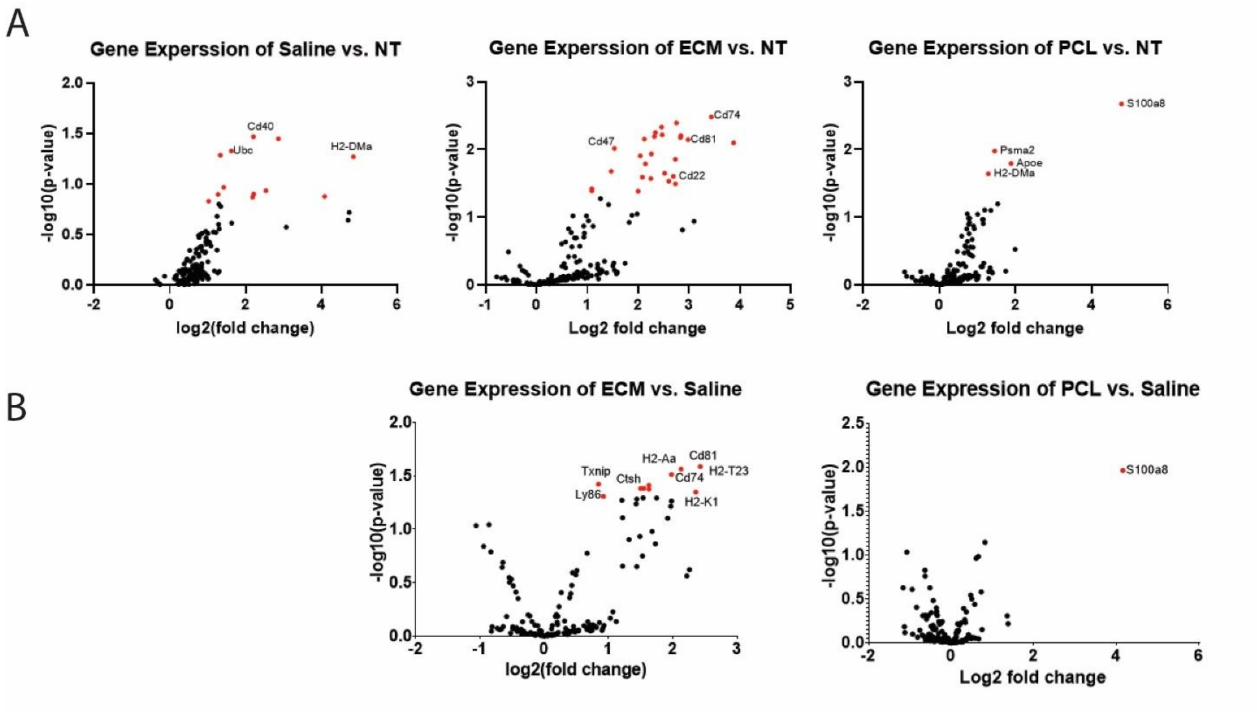

Fig S7. Splenic B cells gene expression. (A) Gene expression volcano plots of expressed genes compared to no treatment (NT). **(B)** Gene expression volcano plots of ECM and PCL compared to Saline. Red indicates statistically significance compared to NT, *p<0.05 in A, compared to Saline in B.

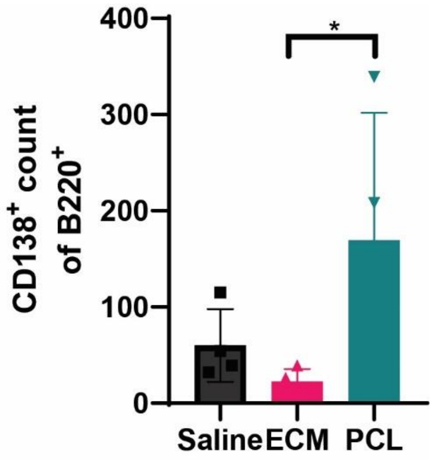

Fig 8. PCL induces CD138$^+$ B220$^+$ cells in the quad tissue. CD138$^+$B220$^+$ cell population in quad tissue at 3 weeks post-injury. Data are means ± SD, n=4, Two-way ANOVA with subsequent multiple comparison testing [* p<0.05].

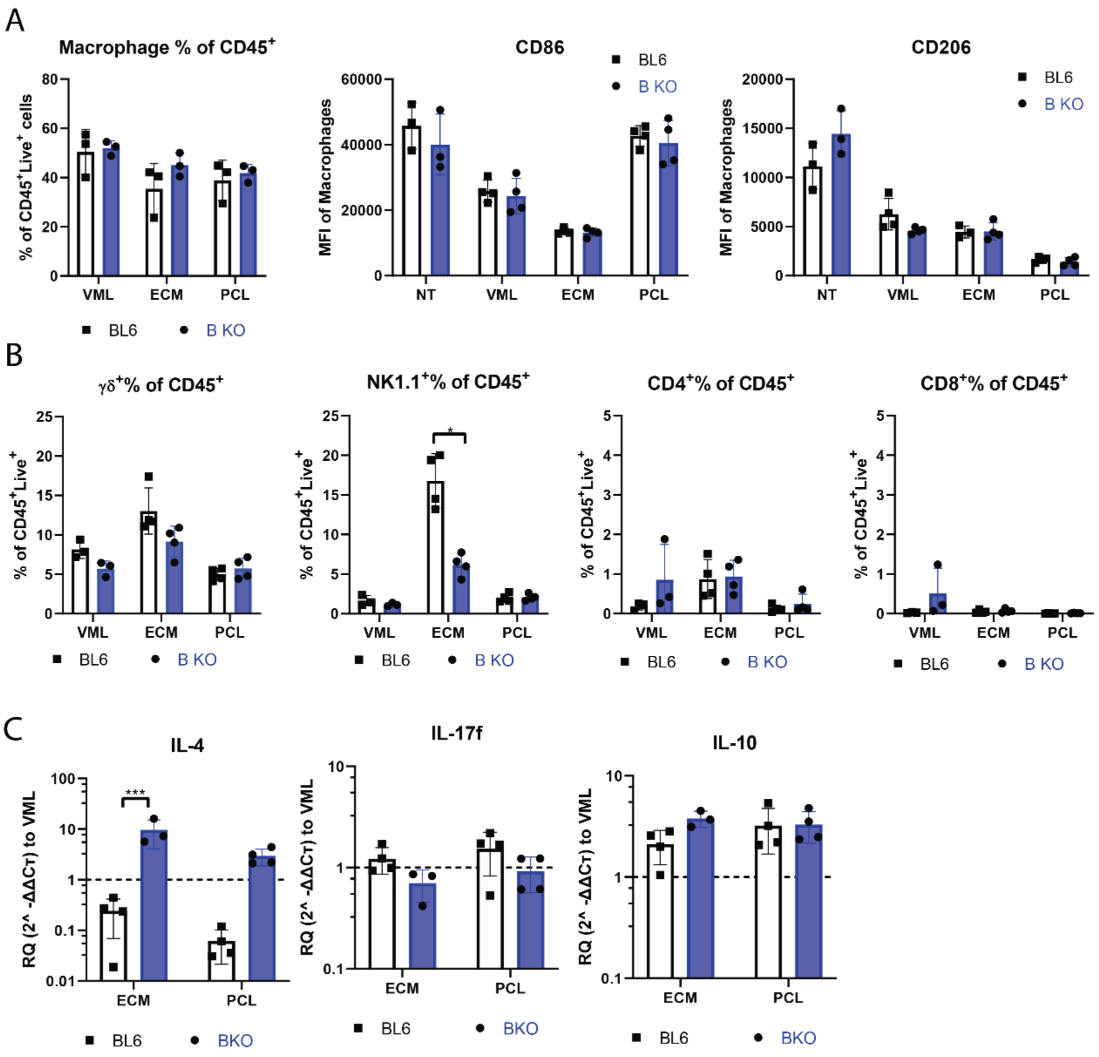

Fig S9. Cell and genetic differences between MuMt^{-} mice and BL6 mice. (A-B) Flow cytometry analysis of **(A)** macrophages with quantification of expression of CD86 and CD206 at 1 week post -injury **(B)** CD4, CD8 T cells, NK1.1 and $\gamma\delta$ T cells as a percentage of CD45^{+}Live^{+} cells in quad at 1-week post-injury. **(C)** Gene expression analysis of*il4*, *il17f,* and *il10* at 6-weeks post injury in the iLN. Data are means ± SD, n=4, Two-way ANOVA with subsequent multiple comparison testing [* p<0.05].

Table S1. Antibodies for B cell flow cytometry panel				
**Fluorophore**	**Marker**	**Clone**	**Catalog**	**Manufacturer**
Brilliant Violet 421™	CD19	6D5	115537	Biolegend
BV605	CD45	30-F11	103155	Biolegend
BV711	CD11b	M1/70	101242	Biolegend
AF488 (FITC)	CD3	17A2	100210	Biolegend
PE	CD138	281-2	142503	Biolegend
PE/Dazzle 594	I-A/I-E	M5/114.15.2	107648	Biolegend
PE-Cy7	B220	RA3-6B2	103221	Biolegend
APC	CD27	LG-3A10	124211	Biolegend
AF700	CD5	53-7.3	100635	Biolegend
eFluor780	Viability		65086514	ThermoFisher

Table S1. B cell flow cytometry staining panel.

Table S2. Antibodies for FACS (B cell sorting)				
**Fluorophore**	**Marker**	**Clone**	**Catalog**	**Manufacturer**
Brilliant Violet 421™	CD19	6D5	115537	Biolegend
BV605	CD45	30-F11	103155	Biolegend
AF488 (FITC)	CD3	17A2	100210	Biolegend
eFluor780	Viability		65086514	ThermoFisher

Table S2. B cell sorting antibodies for Fluorescent Assorted Cell Sorting.

Table S3. Antibodies for flow cytometry (T cell panel)				
**Fluorophore**	**Marker**	**Clone**	**Catalog**	**Manufacturer**
BV421	FoxP3	MF-14	126419	Biolegend
V500	CD45	30-F11	561487	BD Biosciences
BV605	NK1.1	PK136	108739	Biolegend
BV711	CD8	53-6.7	100748	Biolegend
AF488 (FITC)	CD3	17A2	100210	Biolegend
PerCP-Cy5.5	CD19	6D5	115534	BioLegend
PE	IL-4	11B11	504103	Biolegend
PE-Dazzle 594	I-A/I-E	M5/114.15.2	107648	Biolegend
PE-Cy7	CD4	GK1.5	100422	Biolegend
APC	IFNγ	XMG1.2	505810	Biolegend
AF700	IL-17a	TC11-18H10.1	506914	Biolegend
eFluor780	Viability		65086514	ThermoFisher

Table S3. Flow cytometry antibody panel for T cell characterization.

Table S4. Murine TaqMan gene expression primers			
**Primer**	**Assay ID**	**Catalog #**	**Manufacturer**
IL-4	Mm00445259_m1	4331182	ThermoFisher
IL-17f	Mm00521423_m1	4331182	ThermoFisher
IL-17a	Mm00439619_m1	4351370	ThermoFisher
IL-6	Mm00446190_m1	4331182	ThermoFisher
Aicda	Mm01184115_m1	4331182	ThermoFisher
Jchain	Mm00461780_m1	4331182	ThermoFisher
p21	Mm01332263_m1	4351370	ThermoFisher
TGF-β	Mm01178820_m1	4331182	ThermoFisher
IL-23	Mm00518984_m1	4331182	ThermoFisher
IL-10	Mm01288386_m1	4331182	ThermoFisher
S100a4	Mm00803372_g1	4351370	ThermoFisher
**α**SMA (ACTA2)	Mm00725412_s1	4331182	ThermoFisher
Col1a1	Mm00801666_g1	4331182	ThermoFisher
Col3a1	Mm01254477_m1	4351370	ThermoFisher

Table S4. Murine TaqMan gene expression primers.
